# Correlation between superior humeral head migration and proximal long head of biceps tendon pathology in patients with and without rotator cuff tears using magnetic resonance imaging and radiography

**DOI:** 10.1016/j.jseint.2022.06.003

**Published:** 2022-07-03

**Authors:** Josh Rattee, Laura Sims, David A. Leswick, Haron Obaid

**Affiliations:** a Faculty of Medicine, University of Saskatchewan, Saskatoon, SK, Canada; b Division of Orthopedic Surgery, Department of Surgery, Faculty of Medicine, University of Saskatchewan, Saskatoon, SK, Canada; c Department of Medical Imaging, Faculty of Medicine, University of Saskatchewan, Saskatoon, SK, Canada

**Keywords:** Rotator cuff, Long head of biceps tendon, Humerus, MRI, Radiographs, Acromiohumeral distance

## Abstract

**Background:**

The biomechanical role of the proximal long head of the biceps tendon (PLHB) in glenohumeral joint stability remains controversial. This retrospective study aims to correlate between humeral head migration and PLHB pathology in patients with and without rotator cuff tendon tears using imaging.

**Methods:**

Seventy-nine patients who underwent 3T magnetic resonance imaging of the shoulder were retrospectively reviewed. Imaging findings were documented by a fellowship-trained musculoskeletal radiologist. PLHB tendon diameter change, contour irregularity, and signal intensity change were assessed. Rotator cuff status was given a binary assignment of intact vs. torn. Radiographs were used for measurement of the acromiohumeral distance and a cutoff value of 7 mm was set as a lower limit of normal.

**Results:**

In the cohort of 79 shoulders, 41.8% (33/79) of patients had intact PLHB tendon and rotator cuff, 26.6% (21/79) demonstrated isolated PLHB tendon pathology, 13.9% (11/79) demonstrated isolated rotator cuff tears, and 17.7% (14/79) demonstrated concomitant PLHB tendon pathology and rotator cuff tears. Acromiohumeral distance was preserved in 97.0% (32/33) of patients with intact PLHB tendon and rotator cuff, 28.6% (6/21) of patients with isolated PLHB tendon pathology, 81.8% (9/11) of patients with isolated rotator cuff tears, and 14.3% (2/14) of patients with concomitant PLHB tendon pathology and rotator cuff tears (*P* < .0001).

**Conclusion:**

Results of this study have shown that a statistical correlation was present between superior humeral head migration and PLHB tendon pathology with or without rotator cuff tears, compared to rotator cuff pathology alone. Findings suggest that intact PLHB tendon plays an important role in glenohumeral stability.

Glenohumeral joint stability is a multifactorial process that relies on a delicate balance between several static and dynamic stabilizers. The shoulder capsule, glenoid labrum, glenohumeral ligaments, glenoid cavity, and humeral head in conjunction with the muscular stabilizers allow for a wide range of motion in the shoulder joint.^3^ Stability through this wide range of motion partially depends on the relationship between the humeral head and scapula, and it has been suggested that superior migration of the humeral head is a mechanical factor in the etiology of rotator cuff tears.^8^

The role of the proximal long head of the biceps (PLHB) tendon is a long-debated topic.^6^ Despite often being referred to as a pain generator,^4^ there are numerous biomechanical studies suggesting the PLHB tendon to be a depressor of the humeral head that confers passive stability to the shoulder joint.^1^^,^^10^^,^^19^ Specifically, there is cadaveric evidence suggesting that complete PLHB tendon disruption allows for significant superior migration of the humeral head and reduction in acromiohumeral distance.^17^ Although cadaveric studies have shown some evidence to support the biomechanical role of the PLHB, these studies are limited in assessing superior humeral head migration due to unbalanced deltoid muscle contraction, which is seen on imaging in living humans.^2^ Furthermore, there is limited radiologic research concerning the relationship between PLHB tendon pathology and humeral head position. The primary aim of the study is to correlate between superior humeral head migration and PLHB pathology. Secondary aim is to assess whether or not the status of the PLHB has any effect on superior humeral migration in patients with or without rotator cuff pathology using magnetic resonance imaging (MRI) and radiographs.

## Materials and methods

### Study design and protocol

Ethical approval for this study was obtained from the institutional ethics board. This study was completed in accordance with the World Medical Association Declaration of Helsinki. A single-center retrospective analysis of routine shoulder MRI scans was completed. Patients were identified using Montage (Nuance mPower, Burlington, MA, USA) from January 2017 to April 2021. Patient information included in the study was made anonymous by using a master list. The patient identifying information was converted into a unique study identification number and included on this list.

### Patients

Adult patients aged 18-50 who underwent radiographic examination and routine noncontrast shoulder MRI protocol using the 3T scanner from 2015 to 2021 were considered eligible for inclusion. Patients were excluded if they had a history of prior surgery, trauma, fracture, arthropathy (inflammatory, crystalline, or infectious), calcific tendinopathy, bone and soft tissue tumors, or presence of artifact on imaging. Patients were also excluded if they lacked corresponding radiographs to match the results. As all collected patient information was obtained from the picture archiving and communication system, demographics regarding height and body mass index were not documented.

### Imaging

Routine shoulder MRI protocol was performed on a Siemens 3T scanner (MAGNETO Skyra; Siemens Healthcare, Erlangen, Germany) using Software Numaris/4, Version Syngo MR E11 and Siemens 16 channel shoulder coil. The imaging protocol is described in Table I. All images were reviewed on Picture Archiving and Communication System (Philips IntelliSpace PACS 4.4.541.5) and displayed on Coronis Fusion 6-megapixel LED Barco monitors (MDCC-6230; Barco NV, Kortrijk, Belgium). Shoulder radiographs included anteroposterior (AP), internal rotation, transscapular, and axillary views.

**Table I:** Sequences and parameters for the routine shoulder MRI protocol using 3T scanner (Siemens MAGNETO Skyra).

Sequence	TR (ms)	TE (ms)	Slice thickness (mm)	Matrix (mm)	Field of view (mm)
Axial MEDIC	540	14	3.0	0.3 × 0.3 × 3.0	160
Coronal oblique T2	4000	78	3.0	0.4 × 0.4 × 3.0	160
Axial proton density fat saturated	3200	40	3.0	0.4 × 0.4 × 3.0	160
Coronal oblique proton density fat saturated	2500	29	3.0	0.4 × 0.4 × 3.0	160
Sagittal oblique proton density fat saturated	3070	29	3.0	0.4 × 0.4 × 3.0	160
Sagittal oblique T1	804	21	3.0	0.4 × 0.4 × 3.0	160

*MEDIC*, multiple echo data image combination; *TE*, time to echo; *TR*, time to repeat.

### Data collection

Eligible patient charts were reviewed and patients’ age, sex, and indication for imaging were documented. MRI findings were documented by a fellowship-trained musculoskeletal radiologist as per previously published studies.^3^^,^^9^ A simple binary assignment of normal vs. pathological was assigned to the PLHB tendon based on findings of diameter change, contour, signal changes, tearing, and dislocation. PLHB tendon findings were deemed pathological if it appeared bulbous, irregularly marginated, high in signal intensity, or situated outside the bicipital groove. Similarly, rotator cuff (RC) status was recorded and given a binary assignment of intact vs. torn. RC tendons identified as torn were further subdivided into partial vs. full thickness tears. Corresponding radiographs obtained prior to MRI were used to measure acromiohumeral distance and a binary assignment of normal vs. reduced was assigned. The acromiohumeral distance was defined as the shortest distance between the inferior cortex of the acromion and the humeral head with the shoulder in neutral rotation on AP view. A measurement of <7 mm was considered abnormal.^13^

### Statistical analysis

Separate datasets were created and analyzed with categorical values expressed as counts. The first dataset compared PLHB tendon status and RC status to acromiohumeral distance measurement using a chi-squared test (Table II). The second compared PLHB tendon status and partial thickness RC tears to acromiohumeral distance measurement using a Fisher’s exact test (Table III). The third dataset compared PLHB tendon status and full-thickness RC tears to acromiohumeral distance and was also analyzed using a Fisher’s exact test (Table III). Statistical analysis was completed using Prism version 9.1.2 (GraphPad biostatistics software; GraphPad, San Diego, CA, USA).

**Table II:** PLHB tendon pathology and rotator cuff status vs. acromiohumeral distance.

Tendon status	Acromiohumeral distance	
	Normal (7 mm)	Reduced
Normal PLHB tendon	32	1
Intact rotator cuff		
Pathological PLHB tendon	6	15
Intact rotator cuff		
Normal PLHB tendon	9	2
Torn rotator cuff		
Pathological PLHB tendon	2	12
Torn rotator cuff		

*PLHB*, proximal long head of the biceps.

**Table III:** PLHB tendon pathology and rotator cuff status further subdivided into partial or complete tear vs. acromiohumeral distance.

Tendon status	Acromiohumeral distance	
	Normal (>7 mm)	Reduced
Normal PLHB tendon	7	1
Partially torn rotator cuff		
Pathological PLHB tendon	2	4
Partially torn rotator cuff		
Normal PLHB tendon	2	1
Completely torn rotator cuff		
Pathological PLHB tendon	0	8
Completely torn rotator cuff		

*PLHB*, proximal long head of the biceps.

## Results

A total of 273 cases were identified from January 2017 to April 2021. After excluding those with prior surgery, trauma, fracture, arthropathy, or tumor, a total of 105 patients were deemed eligible. An additional 26 participants were excluded due to lack of radiographs for a final cohort of 79. Females comprised 32.9% (26/79) of study participants. The mean participant age was 37.7 years.

In the cohort of 79 shoulders, 41.8% (33/79) demonstrated intact PLHB and RC, 26.6% (21/79) demonstrated isolated PLHB pathology, 13.9% (11/79) isolated demonstrated RC tears, and 17.7% (14/79) demonstrated concomitant PLHB pathology and RC tears. Of the 25 total RC tears, 11 were full thickness tears and 14 were partial thickness tears.

**Figure 1:**
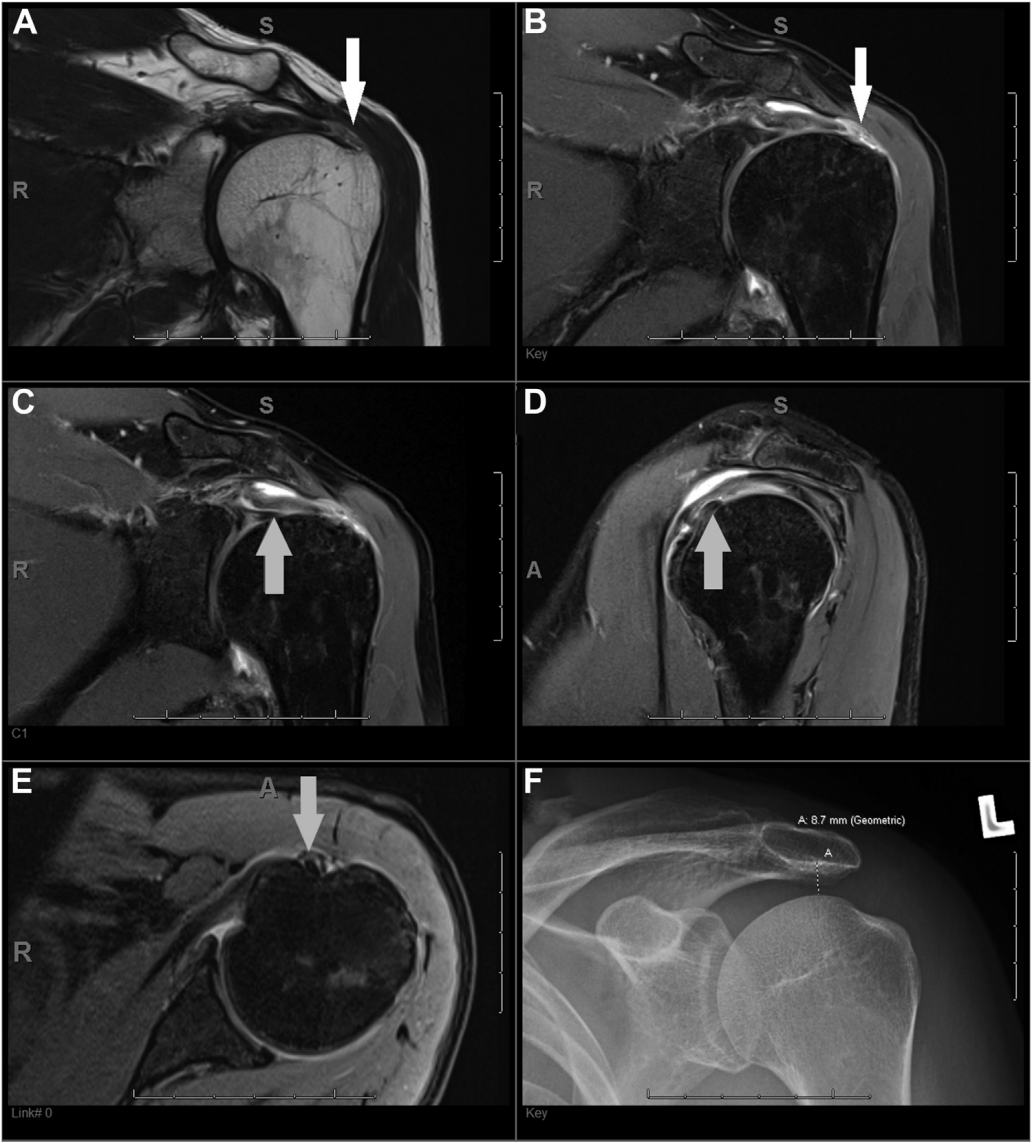
A 3T MRI scan of the left shoulder joint on a 35-year-old male patient using routine protocol. (**A**) Coronal T2-weighted image and (**B**) coronal proton density fat saturated image demonstrating a full thickness tear of the supraspinatus tendon insertion (*white arrows*). (**C**) Coronal proton density fat saturated image, (**D**) sagittal proton density fat saturated image, and (**E**) axial proton density fat saturated image demonstrating an intact biceps anchor and pulley within the bicipital groove (*gray arrows*). (**F**) A frontal radiograph of the left shoulder showing a maintained acromiohumeral distance (8.7 mm). *MRI*, magnetic resonance imaging.

**Figure 2:**
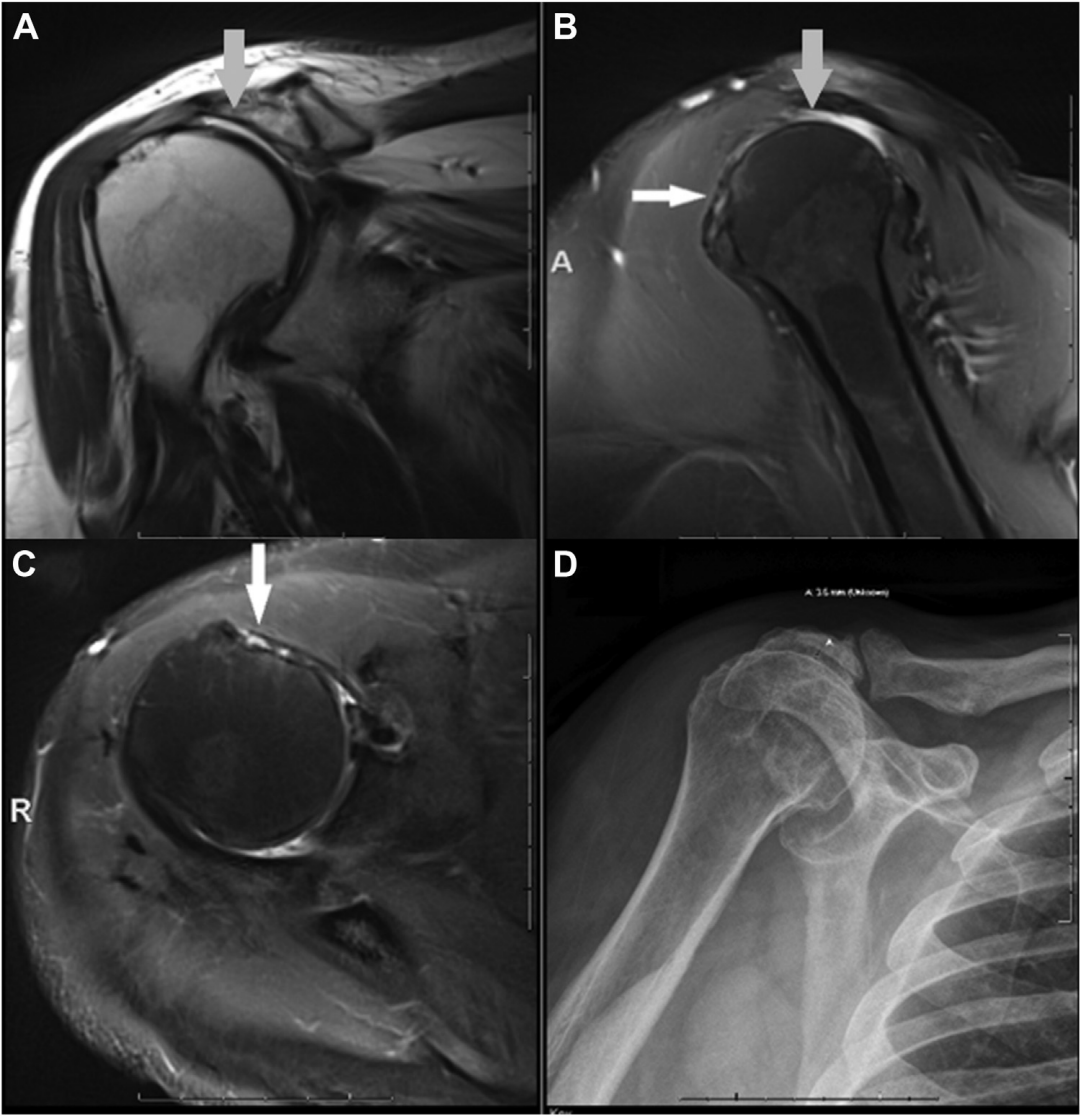
A 3T MRI scan of the right shoulder on a 48-year-old female patient using routine protocol. (**A**) Coronal T2-weighted image and (**B**) sagittal proton density fat saturated image demonstrating complete full thickness tears of the supraspinatus tendon (*gray arrows*). (**B**) Sagittal proton density fat saturated image and (**C**) axial proton density fat saturated image demonstrating absent PLHB tendon in the bicipital groove in keeping with full thickness tear (*white arrows*). (**D**) Corresponding frontal radiograph shows superior humeral head migration and narrowing of the acromiohumeral distance (3.5 mm). *MRI*, magnetic resonance imaging.

**Figure 3:**
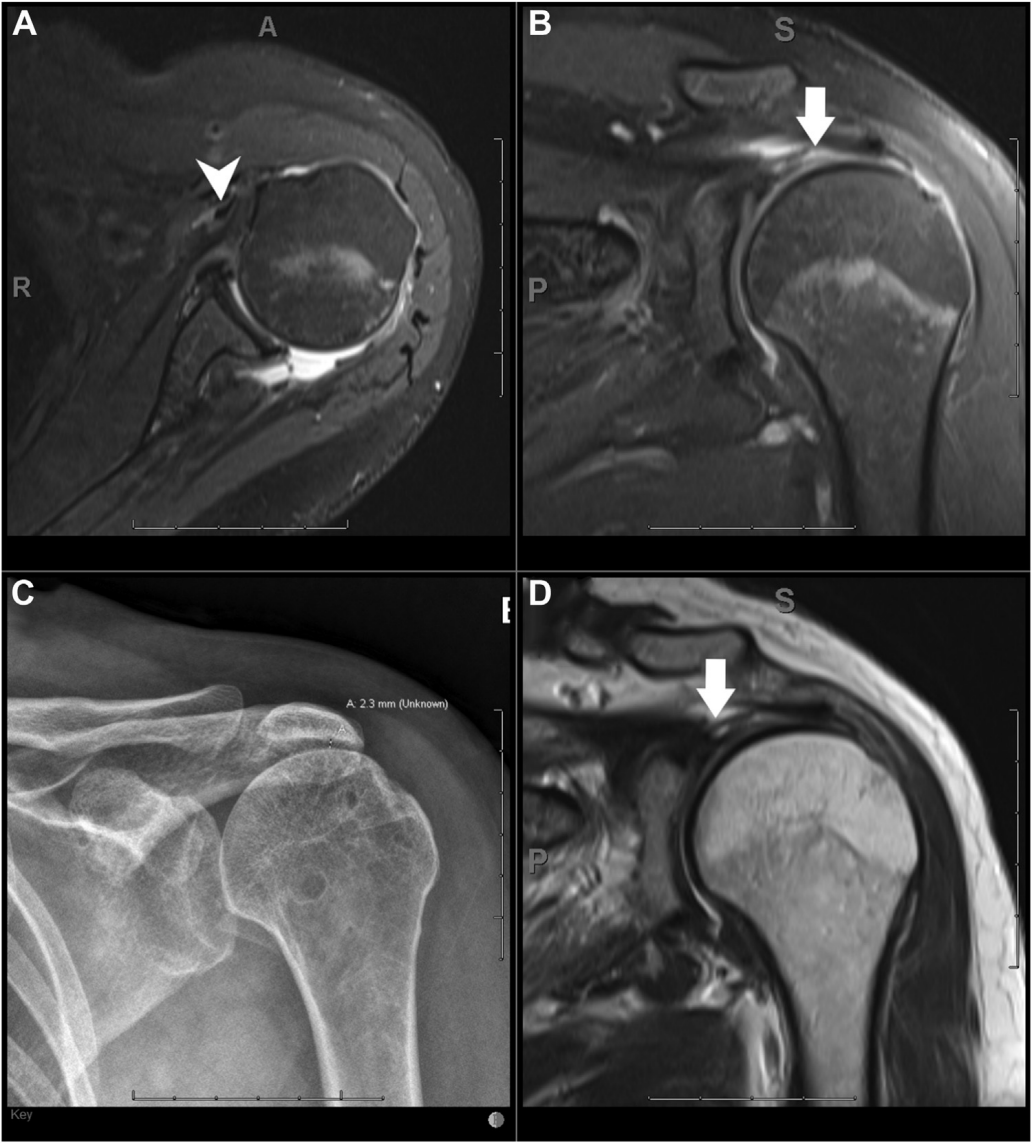
A 3T MRI scan of the left shoulder on a 40-year-old male patient using routine protocol. (**A**) Axial proton density fat saturated image demonstrating medial dislocation of the PLHB (*white arrow head*) and empty bicipital groove. (**B**) Coronal proton density fat saturated image and (**C**) coronal T2 weighted image showing complete full thickness tear of the supraspinatus tendon (*white arrows*). (**D**) A corresponding frontal radiograph of the left shoulder showing superior humeral head migration with narrowing of the acromiohumeral distance (2.3 mm). *MRI*, magnetic resonance imaging; *PLHB*, proximal long head of the biceps.

The acromiohumeral distance was narrowed (<7 mm) in 3% (n = 1/33) of patients with intact PLHB and RC, 71.4% (n = 15/21) of patients with isolated PLHB tendon pathology, 18.2% (n = 2/11) of patients with isolated RC tears (Fig. 1), and 85.7% (n = 12/14) of patients with concomitant PLHB tendon pathology and RC tears, *P* < .0001 (Figs. 2 and 3). Results are shown in Table II.

In patients with partial-thickness RC tears, the acromiohumeral distance was narrowed in 12.5% (n = 1/8) of patients with a normal PLHB tendon, and 66.7% (n = 4/6) of patients with PLHB tendon pathology, *P* = .09 (Table III). The 2 patients with normal acromiohumeral distance had tendinosis of the PLHB, while the 4 patients with reduced acromiohumeral distance had partial tear (n = 3/4) and full thickness tears (n = 1/4) of the PLHB. Whereas in full-thickness RC tears, the acromiohumeral distance was narrowed in 33.4% (n = 1/3) of patients with a normal PLHB tendon and 100% (n = 8/8) of patients with PLHB tendon pathology, *P* = .05.

## Discussion

The aim of this study is to explore the association between PLHB pathology and narrowing of the acromiohumeral distance in patients with and without rotator cuff pathology using imaging tests. New and existing diagnostic signs have allowed for enhanced investigation of the PLHB tendon and its surrounding structures, making MRI an effective tool in investigating these structural relationships.^20^^,^^22^

There are unique structural properties that allow the PLHB tendon to slide seamlessly within the bicipital groove using the humeral head as a lever.^14^ Repetitive microtrauma is thought to disrupt this structural adaptation and by extension impair shoulder kinematics, resulting in a cascade of shoulder dysfunction ranging from subacromial impingement to rotator cuff tear.^9^^,^^19^

There have been several biomechanical studies demonstrating the PLHB tendon’s role in stabilizing the humeral head on the glenoid,^1^^,^^3^^,^^19^^,^^21^ but far fewer studies in vivo. The first study to validate an in vivo stabilizing effect using radiographs was performed by Warner and McMahon in 1995.^21^ They analyzed 7 patients undergoing RC surgery who had MRI documented complete disruption of the PLHB tendon. Using measurements obtained from AP radiographs, they found that complete rupture of the PLHB tendon was associated with significant superior translation of the humeral head and theorized that it may contribute to the development of impingement syndrome, although this was limited by sample size. Since then, very few radiologic studies have been conducted in this area. One study performed by Giphart et al^5^ used fluoroscopy to determine glenohumeral translation during dynamic movement in patients who had undergone subpectoral biceps tenodesis. Investigators concluded that tenodesis patients had no change in glenohumeral kinematics, but due to a small sample size, statistical analysis was not carried out.

This study has corroborated the observations made by Warner and McMahon with a larger sample size. Our results demonstrated that 71.4% of shoulders with PLHB pathology alone showed statistically significant association (*P* < .0001) with acromiohumeral distance narrowing suggesting that PLHB tendon pathology may contribute to loss of acromiohumeral distance and aberrant humeral head position. Our results also showed acromiohumeral distance narrowing to be more prevalent in patients with concomitant PLHB tendon pathology and RC tears (85.7%). In patients with full-thickness RC tears, the acromiohumeral distance was narrowed in 33.4% of patients with a normal PLHB tendon and 100% (no = 8/8) of patients with PLHB tendon pathology, *P* = .05. A healthy PLHB tendon may, therefore, help prevent humeral head migration in patients with RC tears. It was previously reported that decentralization of humeral head is thought to predispose individuals to RC injury.^8^ The proper gliding motion of the PLHB tendon within the bicipital groove is complex as optimal functioning of the PLHB tendon relies on proper gliding resistance distribution of forces requiring proper elastic deformation and recoiling within the tendon itself.^14^ It is plausible that in the presence of biceps tendon pathology, the tensile demands and biomechanical properties of the PLHB tendon are altered.

Acromiohumeral distance of <7 mm was found to be associated with rotator cuff abnormalities.^18^ The acromiohumeral distance was found to be reliable and reproducible.^7^ A narrowed acromiohumeral distance has been used by surgeons as a predictor of poor outcomes following rotator cuff repair because of association of large cuff tears with fatty degeneration of the rotator cuff musculature.^5^^,^^16^^,^^20^ However, this link between a narrowed acromiohumeral distance and size of rotator cuff tear is not perfect as illustrated by an MRI study showing significant association between narrowed acromial-humeral distance and both RC tear and fatty infiltration of musculature, yet the relationship was not perfect as 10% of patients with narrowed acromial-humeral distance had intact RC.^16^ There was no mention of PLHB in that report.^16^ Additionally, other studies have shown that narrowed acromiohumeral distance by itself is not an independent predictor of RC repair success.^11^^,^^12^^,^^15^ Perhaps the status of PHLB is the confounding factor in the reliability of a narrowed acromiohumeral distance as a predictor of poor postoperative outcomes.

There are limitations to this study that must be considered. This is a retrospective study with potential selection bias as each patient undergoing medical imaging has some level of shoulder pain or dysfunction, which makes it difficult to have a truly asymptomatic healthy control group. Sample size was relatively small. The study was limited to examining the association between acromiohumeral distance, and PLHB and RC pathology rather than a causal relationship.

## Conclusions

The results of this study demonstrated a statistically significant association between superior humeral head migration and PLHB pathology with or without RC tears compared to RC pathology alone suggesting that a healthy PLHB plays an important role in glenohumeral joint superior stability. Preservation of the PLHB during shoulder surgery may be useful to maintain glenohumeral joint stability.

## Disclaimers:

Funding: No funding was disclosed by the authors.

Conflicts of interest: The authors, their immediate families, and any research foundations with which they are affiliated have not received any financial payments or other benefits from any commercial entity related to the subject of this article.
